# Lipid signaling and foam cell formation in atherosclerosis and spinal cord injury

**DOI:** 10.1016/j.jlr.2026.101153

**Published:** 2026-09-21

**Authors:** Guina Tan, Xiaoxin Wang, Jing Chen, Ru Feng, Hongyu Chu, Degang Yang, Jun Li, Mingliang Yang, Jianjun Li, Ying Huang, Feng Gao

**Affiliations:** 1 School of Rehabilitation, Gannan Medical University, Ganzhou City, Jiangxi Province, China; 2 Department of Spinal and Neural Functional Reconstruction, China Rehabilitation Research Center, Beijing, China; 3 Spinal Cord Injury Repair Laboratory, China Rehabilitation Science Institute, Beijing, China; 4 School of Rehabilitation, Capital Medical University, Beijing, China; 5 School of Rehabilitation, Wenzhou Medical University, Wenzhou City, Zhejiang Province, China; 6 Department of Rehabilitation Medicine, Peking University Hospital, Beijing, China; 7 Cheeloo College of Medicine, Shandong University, Jinan, Shandong Province, China

**Keywords:** macrophages, microglia, foam cells, lipid metabolism, atherosclerosis, spinal cord injury

## Abstract

Macrophages and microglia serve as both “scavengers” and “immune sentinels” to maintain tissue homeostasis. However, when the lipid load exceeds their processing capacity, these cells transform into foam cells, thereby driving chronic inflammation and impairing tissue repair. Despite their distinct organ systems, atherosclerosis (AS) and spinal cord injury (SCI) converge on a common pathological cascade—foam cell formation—characterized by “enhanced lipid uptake, impaired cholesterol efflux, disrupted lysosomal/autophagic processing, and ER stress/reactive oxygen species/NLRP3-mediated inflammatory amplification.” This review systematically dissects and compares the molecular mechanisms underlying macrophage foaming in AS and SCI, with a particular focus on the similarities and differences in *ABCA1*/*ABCG1*-mediated cholesterol efflux, lipid droplet autophagy, the ER stress-NLRP3 axis, and nuclear receptor regulatory networks. Furthermore, it explores therapeutic avenues that target lipid metabolic reprogramming, including natural products, biomimetic nanomedicines, and genetic interventions. By establishing a cross-disease framework for lipid homeostasis disruption, this paper challenges traditional organ-centric views and provides a theoretical foundation that may accelerate the development of shared therapeutic strategies for AS and SCI.

Macrophages and microglia are key effector cells of the mammalian innate immune system. They are widely distributed throughout various tissues and contribute to tissue homeostasis by phagocytosing apoptotic cells, clearing pathogens, and removing tissue debris (1, 2). In arterial walls, macrophages maintain vascular homeostasis by clearing modified lipoproteins such as oxidized low-density lipoprotein (ox-LDL) (3); in advanced plaques, nonimmune cells, including vascular smooth muscle cells (VSMCs), also exhibit robust lipid uptake and a foamy phenotypic transformation (4). In the CNS, microglia, infiltrating macrophages, and nonprofessional phagocytes, such as endothelial and glial cells, collectively participate in clearing myelin debris from damaged oligodendrocytes and other injury-derived fragments (5, 6). However, when the lipid load in the microenvironment exceeds the cellular capacity for lysosomal hydrolysis and cholesterol efflux, these scavenger cells may accumulate intracellular lipid droplets, experience ER stress and lysosomal dysfunction, and adopt a pro-inflammatory, foam cell-like phenotype. This phenotype, in turn, drives chronic inflammatory cascades and impairs tissue repair, partly through reactive oxygen species (ROS)-linked NLRP3 inflammasome activation (6, 7, 8).

This process of immune-metabolic dysregulation constitutes a common pathological feature across various disease spectra. Foam cells have long been regarded as a hallmark pathological feature of atherosclerosis (AS). In atherosclerotic lesions, myeloid macrophages and phenotypically transformed VSMCs extensively uptake ox-LDL, leading to an uncompensated accumulation of intracellular cholesterol esters. This lipid-rich, inflammatory, foam-like phenotype is a key factor driving plaque progression and instability (9, 10). However, a growing body of research indicates that similar foam cell-like phenomena are also observed in spinal cord injury (SCI) and other demyelinating conditions. In the injured CNS, a complex lipid-rich microenvironment composed of myelin debris from degraded oligodendrocytes and non-myelin-derived tissue fragments (5) is extensively phagocytosed by infiltrating macrophages, resident microglia, and other phagocytic cells. This leads to the progressive accumulation of intracellular lipid droplets, triggering a ROS-NLRP3 inflammasome-mediated inflammatory cascade, and ultimately resulting in impaired remyelination. The ROS-NLRP3 inflammasome cascade is linked to impaired myelin regeneration (11). This suggests that foam cell formation is not unique to AS, but may represent a shared metabolic-immune phenotype linking focal vascular inflammation with secondary CNS neuroinflammation (Fig. 1).

**Fig. 1:**
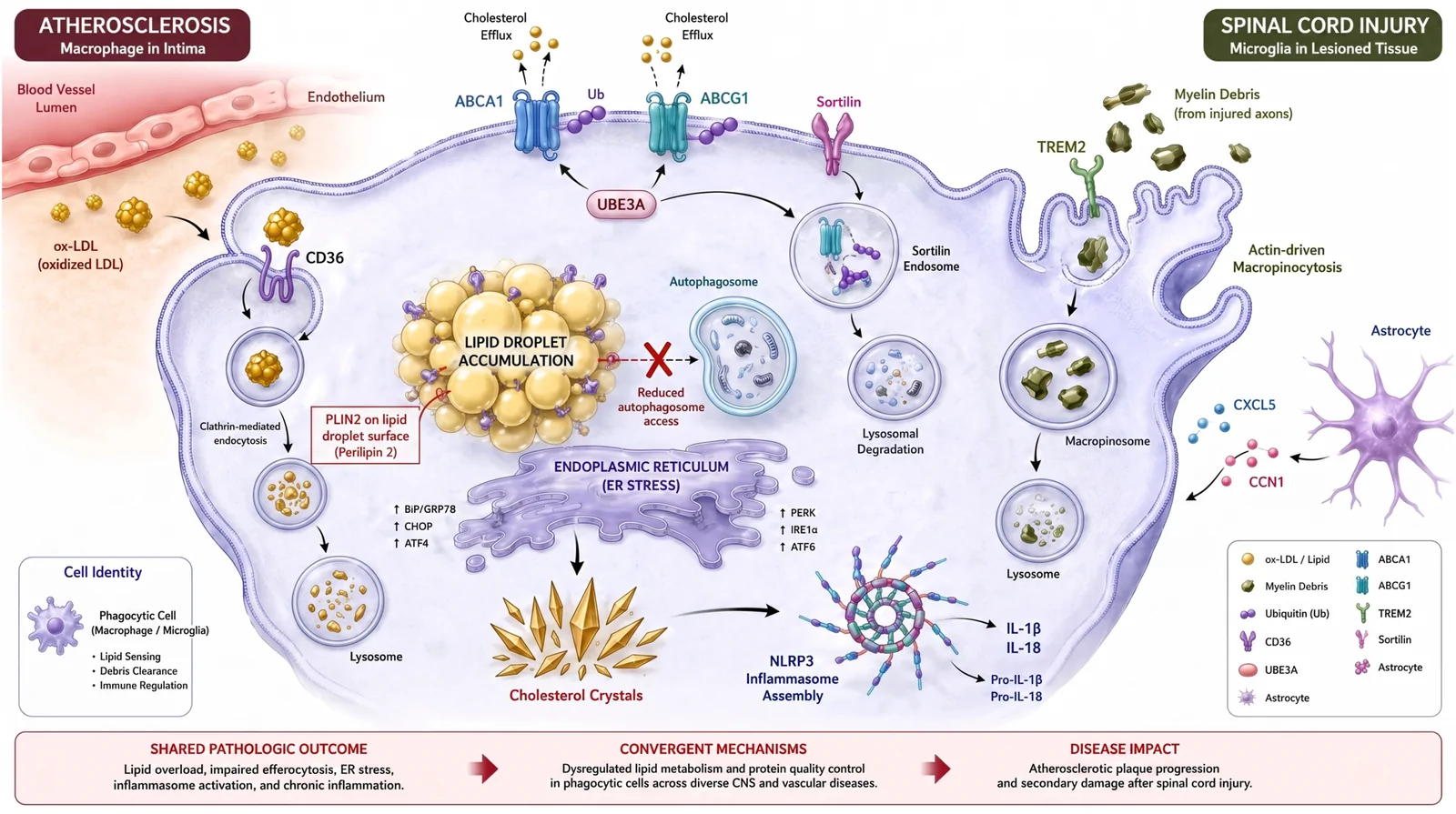
The convergent molecular interplay of lipid homeostasis dysregulation and foam cell formation in atherosclerosis and spinal cord injury. Left, peripheral macrophage-driven ox-LDL uptake via CD36 versus right, central microglial myelin debris clearing via TREM2 and actin-driven macropinocytosis. Internalized lipid overload converges on the shared stagnation of the lipophagy-lysosome axis (enforced by the PLIN2 barrier) and precipitates ER stress-dependent cholesterol crystallization, which ultimately drives NLRP3 inflammasome activation and protein quality control failure via UBE3A/Sortilin-mediated ABCA1/ABCG1 degradation. ABC, ATP-binding cassette; CD, cluster of differentiation; ox-LDL, oxidized low-density lipoprotein; TREM2, triggering receptor expressed on myeloid cells 2.

To counteract lipotoxic damage caused by excessive sterol accumulation, reverse cholesterol transport (RCT) and intracellular lipid efflux pathways provide key protective mechanisms that limit cellular foaming. This network is primarily mediated by the ATP-binding cassette (ABC) transporters ABCA1 and ABCG1, which maintain cellular lipid homeostasis by exporting intracellular lipids to lipid-depleted apolipoproteins or mature HDL, respectively (12, 13, 14). These proteins not only determine the efficiency of cholesterol clearance by macrophages and smooth muscle cells in the peripheral arterial wall (9, 10), but their core efflux cascade also contributes to the resolution of neuroinflammation in central tissues such as the brain and spinal cord (15). In the CNS, ABCA1-mediated lipid efflux is closely linked to the function of the endogenous apolipoprotein E (APOE), which acts as a key lipid acceptor and transporter (16, 17, 18). Thus, impaired lipid efflux is not merely a metabolic abnormality in peripheral AS, but rather a pathological mechanism that may be relevant across peripheral vascular plaques and CNS trauma (19).

Based on this understanding of lipid homeostasis networks across disease spectra, this paper aims to integrate the molecular mechanisms underlying macrophage/microglia-mediated cellular foaming, including the contributions of phenotypically switched smooth muscle and endothelial cells, in AS and SCI(Fig. 1). We focus on the ABCA1/ABCG1-mediated cholesterol efflux pathway, lipid droplet autophagy, the organelle stress (ER/autolysosome)-NLRP3 inflammasome cascade, and nuclear receptor regulatory networks in both conditions. By establishing a cross-disease pathological framework for conserved lipid homeostasis disturbances, this paper provides a theoretical basis for translational therapies targeting metabolic remodeling in immune cells in both AS and SCI.

## The classical molecular network governing macrophage cholesterol efflux and intracellular sterol homeostasis

Under physiological conditions, macrophage cholesterol efflux is a tightly coordinated, multipathway cascade that spans multiple organelles, including lysosomal egress, intracellular trafficking, and plasma membrane (PM) efflux. At the PM, the core transport pumps of this efflux cascade are primarily mediated by the ABCA1 and ABCG1. Specifically, ABCA1 utilizes ATP hydrolysis to drive the efflux of intracellular free cholesterol (Chol) and phosphatidylcholine to lipid-poor apolipoproteins. In peripheral tissues, the primary acceptor is apoA-I, while in the CNS, ABCA1 can additionally use endogenously secreted APOE to initiate the formation of nascent discoidal pre-β-HDL-like particles (13, 20, 21). Subsequently, ABCG1 acts as a central transport hub, promoting the efflux of additional cholesterol to more mature HDL particles. The nascent HDL particles are then remodeled into mature spherical HDL through the esterification of cholesterol by LCAT (22). These two transporters exhibit a functionally complementary and partially compensatory relationship at the PM surface. However, the efficiency of this membrane-bound efflux is not solely determined by the expression levels of the transporters themselves. Instead, it is highly dependent on the directed transport and dynamic distribution of lipid substrates within the intracellular space, including trafficking from endolysosomal stores to the cell surface (23, 24). Prior to the initiation of the membrane-bound efflux cascade, cells rely heavily on a sophisticated intracellular transport network and signal sensors, which constitute a critical upstream node in the orchestration of cholesterol homeostasis (25).

### Intracellular sterol spatial shuttling and upstream cascades of the plasma membrane signaling network

Cholesterol does not accumulate statically within cells but instead flows dynamically between the PM, the endosomal-lysosomal system, the ER, and lipid droplets (Fig. 2). In macrophages, cholesterol is acquired not only through intracellular redistribution but also through the uptake of modified lipoproteins via scavenger receptors, including CD36、scavenger receptor A (SR-A) and LOX-1, followed by endosomal-lysosomal processing. This dynamic equilibrium is maintained by multiple sterol transporters and intracellular sensors.

**Fig. 2:**
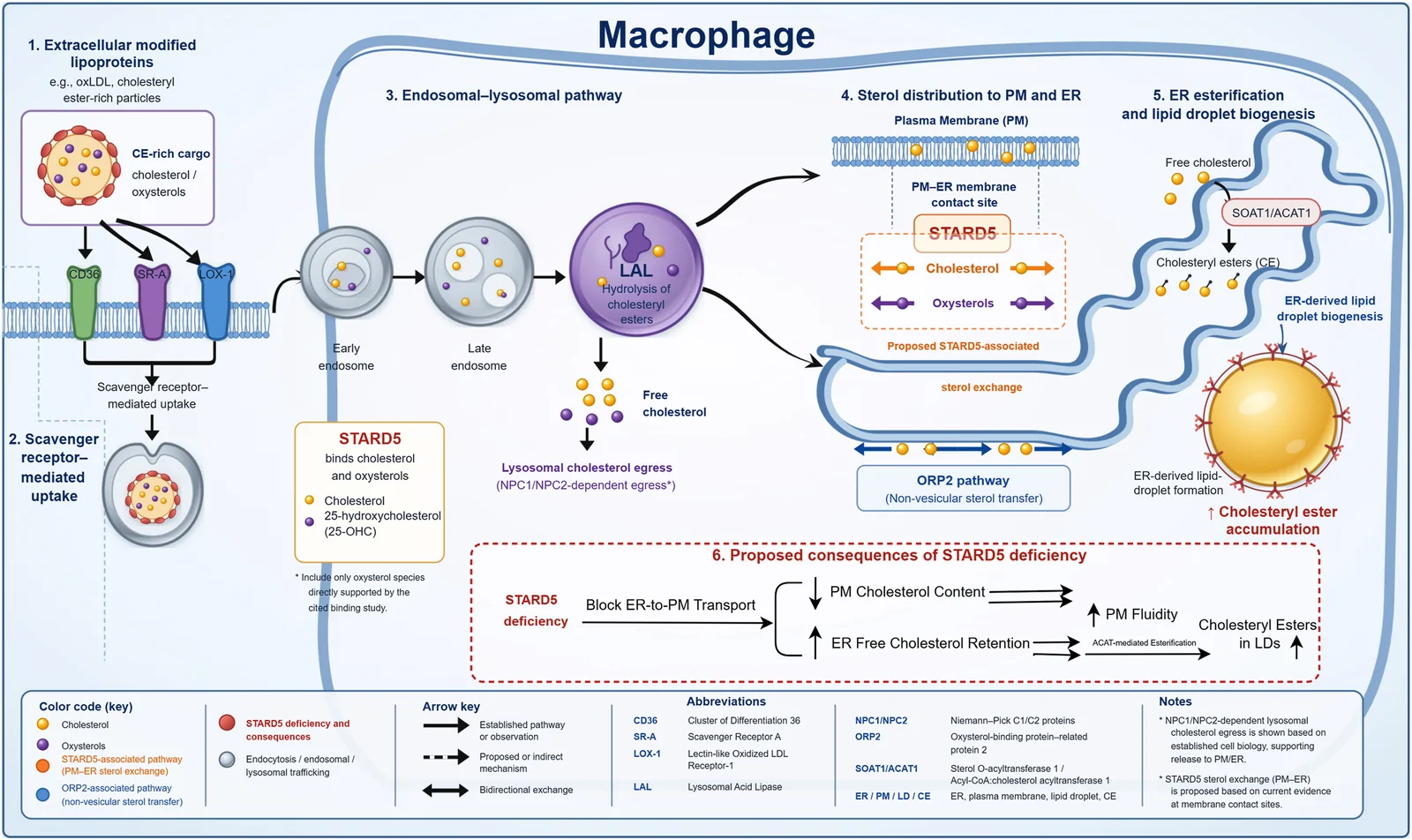
Schematic overview of cholesterol flow between major cellular organelles and the key proteins involved. Organelles depicted include the plasma membrane (PM), endosome-lysosome system, ER, and lipid droplets (LDs). Non-vesicular transport proteins ORP2 and StarD5 mediate cholesterol shuttling between organelles. Lysosomal processing involves lipophagy, in which lipid droplets are delivered to lysosomes for hydrolysis by lysosomal acid lipase (LAL). Perilipin 2 (PLIN2) acts as a physical barrier that limits lipid droplet degradation. Arrows indicate the direction of cholesterol flow. Color code: orange for transport proteins; purple for lysosomal enzymes and processing. ORP2, oxysterol-binding protein-related protein 2.

Among the sterol transporters that contribute to this dynamic equilibrium, oxysterol-binding protein-related protein 2 (ORP2) has been identified as a nonvesicular regulator of cholesterol transport in macrophages. ORP2 can facilitate the transport of free cholesterol from intracellular reservoirs, such as lysosomes, to the PM (23) (Fig. 2). This spatial delivery may contribute to PM cholesterol homeostasis and provide substrate for cholesterol efflux mediated by ABCA1 and ABCG1 at the cell surface. Furthermore, ORP2 has been shown to interact with LXRα and promote its nuclear localization, accompanied by increased expression of ABCA1 and ABCG1, thereby enhancing cholesterol efflux and inhibiting foam cell formation (23). These findings suggest that intracellular cholesterol distribution, mediated by non-vesicular transport networks, may represent an upstream regulatory layer that operates prior to large-scale transcriptional reprogramming of the ABCA1/ABCG1 network.

A second non-vesicular sterol transporter operating within this intracellular cholesterol distribution network is StarD5, a member of the STAR-related lipid transfer domain protein family whose expression is transcriptionally upregulated during ER stress (26). Biochemical characterization of purified StarD5 protein using thermodynamic analysis and solution-state NMR spectroscopy has directly demonstrated that StarD5 binds both cholesterol and 25-hydroxycholesterol, establishing its capacity for sterol ligand recognition beyond cholesterol alone (27, 28). This ligand-binding profile raises the possibility that StarD5 may participate in the handling or redistribution of multiple sterol species in cells, although the directionality and intracellular destinations of oxysterol movement remain to be established (24, 27). These in vitro binding data, however, characterize the ligand recognition properties of the isolated protein and do not, in themselves, define the directionality or destination of sterol transfer in intact cells; the functional context of StarD5-mediated sterol transport is instead established by cellular studies of StarD5 expression manipulation.

Cellular studies have established that StarD5 mediates directed non-vesicular cholesterol transfer from the ER to the PM. The functional importance of this ER-to-PM transport activity is demonstrated by bidirectional manipulation of StarD5 expression: StarD5 deficiency results in a significant reduction in PM cholesterol content and accessible PM cholesterol, accompanied by a corresponding increase in PM fluidity consistent with cholesterol depletion at the membrane. Conversely, StarD5 overexpression produces a marked increase in PM cholesterol levels with a reciprocal reduction in ER cholesterol burden, indicating that StarD5 actively redistributes cholesterol from the ER toward the PM under both basal and stress conditions (24) (Fig. 2). The consequences of StarD5 deficiency, however, extend beyond the PM: when ER-to-PM cholesterol export is impaired, cholesterol is retained within the ER, where it becomes substrate for ACAT1-mediated esterification, driving lipid droplet biogenesis; StarD5 deficiency additionally impairs cholesterol efflux, further compromising intracellular sterol balance in macrophages (29). Critically,this mechanistic cascade demonstrates that StarD5 deficiency-induced lipid droplet accumulation arises as a secondary downstream consequence of impaired ER-to-PM sterol export, accompanied by intracellular free cholesterol retention and subsequent ACAT1-mediated esterification, rather than from direct sterol transport by StarD5 into lipid droplets.

Considered together, the evidence from both ORP2 and StarD5 suggests that non-vesicular sterol transport proteins collectively constitute a spatially organized regulatory layer that governs intracellular cholesterol allocation, modulates membrane sterol composition, and ultimately determines the capacity of macrophages to sustain cholesterol efflux and resist foam cell transformation.

### Transcriptional activation of exporter transporter genes by nuclear receptor networks

The PPARγ-LXRα axis is a well-established transcriptional regulatory module in lipid homeostasis in macrophages and microglia. As endogenous sterol sensors, LXRα and LXRβ can be activated by oxidized sterol ligands, thereby inducing the expression of genes such as *ABCA1*, *ABCG1*, and Cyp27a1. This mechanism mediates ApoA-I-dependent cholesterol efflux by macrophages in the peripheral arterial wall (30). In the CNS, this pathway has been suggested to participate in APOE-mediated sterol transfer to microglia, potentially alleviating myelin-derived lipotoxic damage. Upstream PPARγ can amplify this pathway by upregulating LXRα abundance, forming a PPARγ-LXRα-ABCA1/ABCG1 regulatory cascade.

Several natural products and phytochemicals have been reported to activate lipid clearance processes through this pathway. Pomegranate peel polyphenols can reverse lipid accumulation in RAW264.7 macrophages by upregulating LXRα and ABCA1 expression (31). α-Asarone, identified as a major active component in perilla extract, has been shown to promote sterol efflux in cells exposed to ox-LDL via the PPARγ-LXRα axis (32). The polyphenol derivative PGG (1,2,3,4,6-Penta-O-galloyl-β-D-glucose) has been reported to induce expression of both ABCA1 and the scavenger receptor SR-BI (33) Furthermore, mulberry extract has been shown to upregulate ABCA1/ABCG1 and may inhibit p38 MAPK-NLRP3-mediated inflammatory responses (34). These findings suggest that targeting this nuclear receptor network may represent a strategy for restoring cellular lipid homeostasis in both vascular plaques and sites of neural injury.

Although the aforementioned natural products have shown potential in preclinical studies, the clinical translation of traditional LXR agonists remains challenging. Animal studies and some clinical pharmacology reports have indicated that systemic activation of LXR can lead to hepatic steatosis and hypertriglyceridemia, and may cause neutropenia in humans (35, 36). To circumvent this systemic lipotoxicity, the development of highly selective LXRβ agonists (such as the novel benzofuran-2-carboxylic acid derivative E17110 (37)) or the use of antibody-drug conjugates for macrophage-targeted delivery (38, 39) has become a common frontier in both peripheral plaque clearance and central detoxification. For example, the steroidal LXR agonist DMHCA has been reported to alleviate atherosclerotic lesions in *Apoe*-deficient mice without activating the hepatic SREBP1c lipogenic pathway (36).

### Dynamic coupling between intracellular lipid autophagy pathways and transmembrane efflux networks

Lipid droplets are central organelles in foam cells that store fatty acids predominantly in esterified forms, mainly as triacylglycerols and cholesteryl esters, within a hydrophobic core. The core is surrounded by a phospholipid monolayer containing free cholesterol and lipid-droplet-associated proteins. Classic studies have shown that within lipid-overloaded macrophages, lipid droplets are not inert physical reservoirs, but rather can be selectively enveloped and delivered to the interior of lysosomes, where, under the catalytic action of LAL, the hydrolyzed free cholesterol is specifically supplied to ABCA1 for transmembrane efflux (40). This pathway establishes a “lipodroplet–autophagy–lysosome–efflux” spatial cascade transport axis, suggesting that in the pathological progression of related diseases, the imbalance in lipid homeostasis does not stem solely from a single functional defect in cytoplasmic membrane transporters, but rather from a profound pathological functional disconnection between intracellular lysosomal lipolytic processing and transmembrane retrotransport networks.

This mechanism of cellular foaming, driven by impaired autophagy, exhibits a high degree of pathological homology in the progression of vascular plaques and CNS trauma. Whether in macrophages that take up ox-LDL at high rates in peripheral arterial walls or in microglia that extensively phagocytose myelin debris at sites of CNS injury, the efficiency of intracellular lipid clearance is not only constrained by the initial endocytic flux but also highly dependent on the dynamic turnover efficiency of the lipid droplets themselves.

As a key scaffolding protein enveloping the surface of lipid droplets, PLIN2 forms an endogenous physical barrier that limits the lysosomal degradation of lipid droplets (41, 42). Emerging evidence confirms that, within the microenvironment of secondary neuroinflammation, abnormally high expression of PLIN2 physically blocks the contact between autophagosomes and the lipid droplet core, as well as the lysosomal clearance process. This leads to a stagnation in the metabolic turnover of myelin-derived lipids, thereby inducing phagocytes to persistently maintain a pro-inflammatory phenotype characterized by lipid overload (43, 44, 45). Conversely, targeted PLIN2 knockout reactivates intracellular lipid autophagy, improves the local microenvironment at the site of CNS injury, and promotes remyelination (41). These findings further confirm that the key pathological mechanism underlying cross-organ foam cell remodeling is not solely attributable to excessive uptake of exogenous lipids; dysfunction of the endolysosomal autophagy degradation pathway also constitutes a core driver in the chronic inflammatory cascade.

### Posttranslational modification and degradation regulation of the efflux transporters ABCA1 and ABCG1

The steady-state abundance and spatial localization of transmembrane transporters on the PM do not depend entirely on the transcriptional efficiency of upstream nuclear receptors, but are also influenced by posttranslational modification and protein stability regulatory networks. Protein stability regulation has been proposed as a determinant of cholesterol efflux efficiency in macrophages and microglia. Studies have reported that the intracellular sorting protein Sortilin can bind to ABCA1 and mediate its transfer to the lysosomal pathway, potentially accelerating ABCA1 degradation and foam cell formation (46). Conversely, certain natural product compounds have been reported to affect protein stability: piperine may slow ABCA1 turnover by inhibiting calpain-mediated cleavage (47). Falcarindiol has been suggested to possess a dual regulatory mechanism, potentially activating ABCA1 gene transcription and enhancing its resistance to degradation (48). Furthermore, apolipoprotein A-I binding protein has been reported to protect ABCA1 from degradation by promoting the physical coupling of exogenous ApoA-I with ABCA1 (49) (Fig. 3).

**Fig. 3:**
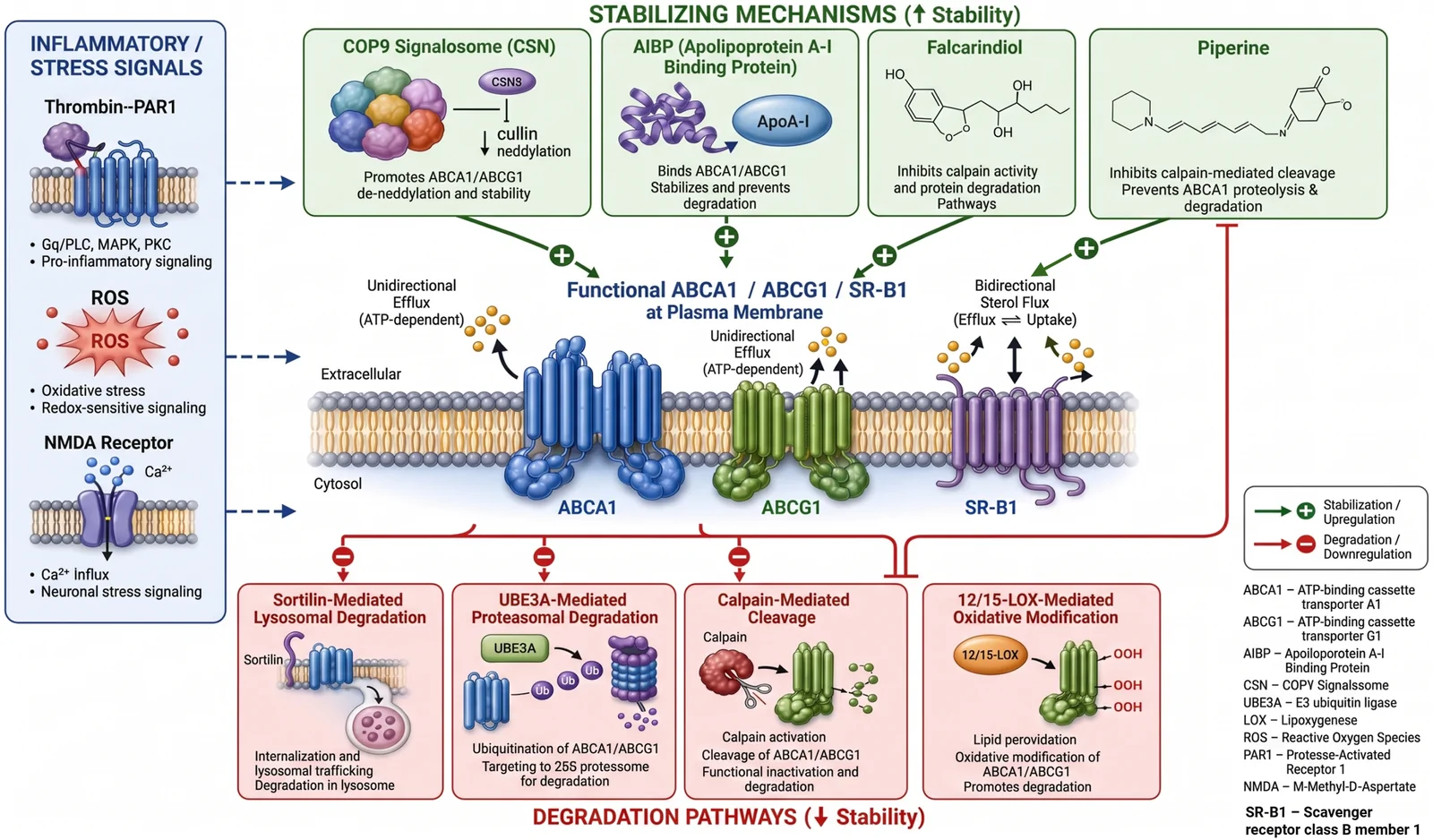
Posttranslational regulation of ABCA1, ABCG1, and SR-B1 stability. Schematic overview of the regulatory network controlling the plasma membrane stability of ABCA1, ABCG1, and SR-B1. Center: functional membrane transporters/receptor, where ABCA1 and ABCG1 mediate cholesterol efflux, whereas SR-B1 supports bidirectional sterol flux. Top: stabilizing mechanisms, including the COP9 signalosome (CSN), apolipoprotein A-I binding protein (AIBP), falcarindiol, and piperine. Bottom: degradation pathways, including Sortilin-mediated lysosomal degradation, UBE3A-mediated proteasomal degradation, calpain-mediated cleavage, and 12/15-lipoxygenase (12/15-LOX)-mediated oxidative inactivation. Left: inflammatory and stress signals that promote transporter/receptor destabilization, including thrombin-PAR1 signaling, reactive oxygen species (ROS), and NMDA receptor overactivation. SR-B1 is included to indicate its role in membrane sterol handling within the same posttranslational regulatory context. Green arrows indicate stabilization; red arrows indicate degradation. ABC, ATP-binding cassette; NMDA, N-methyl-D-aspartate; SR-B1, scavenger receptor class B type 1.

In complex inflammatory and traumatic microenvironments, the homeostatic protective mechanisms of transporter proteins are prone to disruption. The COP9 signalosome has been proposed as a complex that maintains ABCA1 protein stability. However, activation of the thrombin-PAR1 signaling axis, triggered by damage to the vascular endothelial barrier, has been reported to induce phosphorylation of ABCA1, potentially causing its dissociation from the COP9 signalosome and subsequent degradation via the ubiquitin-proteasome pathway (50).

This disruption of protein stability, triggered by microenvironmental stress, may be amplified through a cascade involving receptor activation, oxidative stress, and disruption of protein homeostasis. ROS and G protein-coupled receptor agonists have been reported to induce the structural disintegration of the COP9 complex through H_2_O_2_ production in a xanthine oxidase-dependent manner, potentially driving the degradation of ABCA1 via the Cullin 3 ubiquitin ligase complex (51).

This mechanism of posttranslational depletion, which has been demonstrated in peripheral vascular diseases, may also play a role in CNS inflammation and demyelinating conditions. Studies have reported that excessive activation of NMDA receptors caused by glutamate overload within the CNS injury site can trigger calpain-mediated proteolysis of ABCA1, potentially impairing microglial efflux capacity and promoting a foamy phenotype (52). Furthermore, recent evidence indicates that the E3 ubiquitin ligase UBE3A may act as a negative regulator of ABCA1 stability: UBE3A has been reported to recognize ABCA1 on the cell membrane and direct it to the proteasome for degradation, potentially contributing to foam cell persistence and remyelination defects (53). In addition, elevated activity of 12/15-lipoxygenase (12/15-LOX) has been shown to enhance serine phosphorylation of ABCG1, thereby accelerating its degradation and inactivation (21). Taken together, these findings suggest that blocking the pathological clearance of ABCA1/ABCG1 at the posttranslational level may represent a potential target for restoring lipid retrotransport across organs.

## Macrophage foam cell formation and lipotoxic inflammation in AS

### Imbalance between enhanced lipid uptake and reverse efflux

A central pathological feature of AS is the sustained uptake of modified LDL (particularly ox-LDL) by macrophages derived from peripheral monocytes, local resident macrophages, and phenotypically transformed VSMCs, ultimately leading to the formation of lipid-laden foam cells (54, 55) (Fig. 4). Within this molecular network, CD36 and SR-A primarily regulate lipid uptake, while ABCA1 and ABCG1 govern cholesterol efflux; the imbalance between these two processes is a key determinant of foam cell formation and disease progression (56, 57) (Fig. 4).

**Fig. 4:**
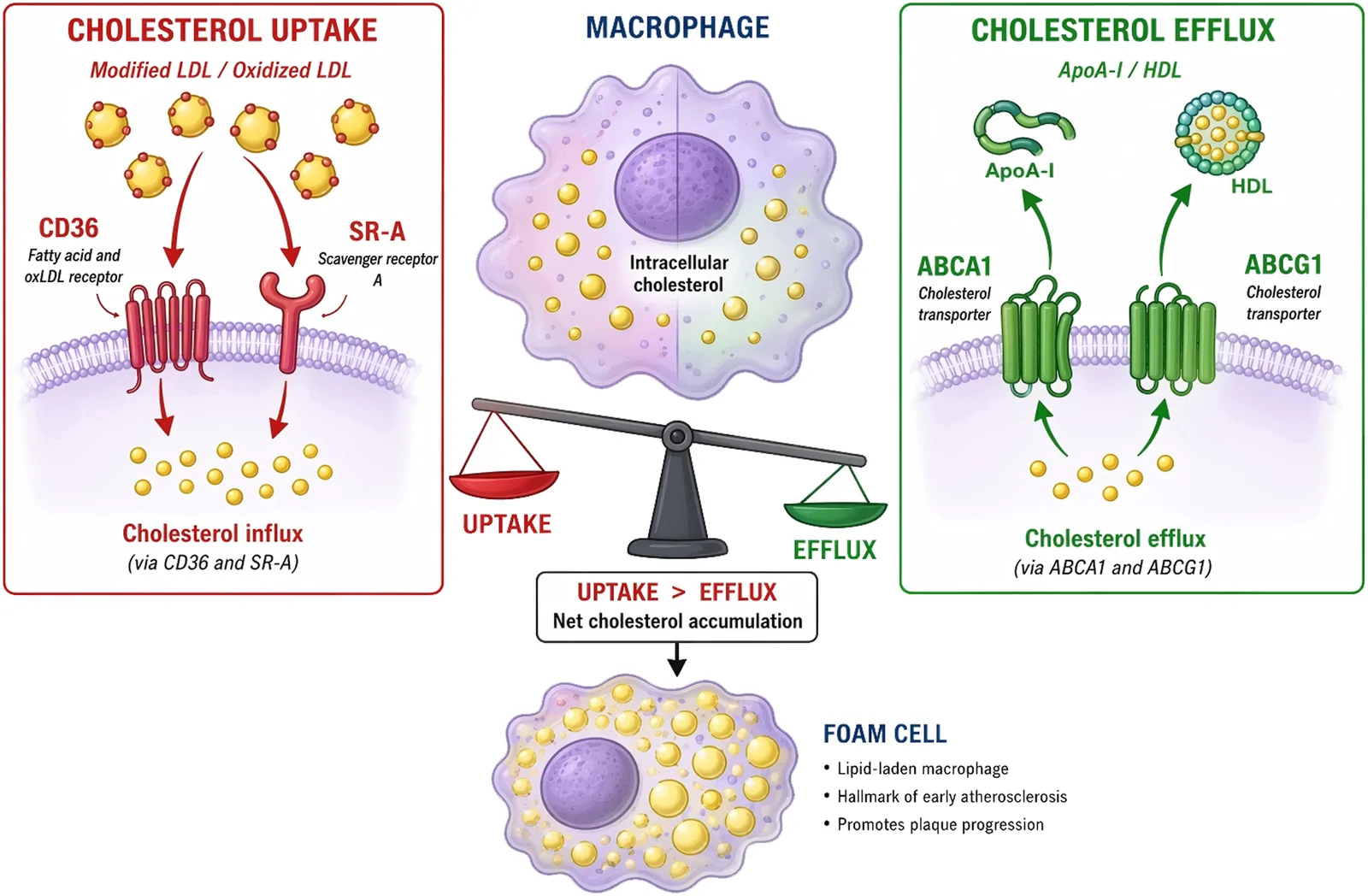
Imbalance between lipid uptake and cholesterol efflux drives foam cell formation. Schematic diagram illustrating the central pathological event in atherosclerosis and spinal cord injury. Left panel: lipid uptake pathways mediated by scavenger receptors CD36 and SR-A, which internalize modified lipoproteins such as ox-LDL. Right panel: cholesterol efflux pathways mediated by ATP-binding cassette transporters ABCA1 and ABCG1, which export cholesterol to extracellular acceptors including ApoA-I, HDL, and APOE. The central balance scale, tilted to the left, indicates that lipid uptake exceeds efflux capacity. Bottom panel: foam cell formation resulting from this imbalance. Color code: red for uptake pathways; green for efflux pathways. ABC, ATP-binding cassette; APOE, apolipoprotein E; CD, cluster of differentiation; ox-LDL, oxidized low-density lipoprotein.

Several inflammatory signaling pathways have been reported to participate in the remodeling of this uptake-efflux balance. The P2Y6 receptor is highly expressed in atherosclerotic plaques and has been suggested to upregulate SR-A expression through the PLCβ/SOCE/CALR cascade, thereby promoting ox-LDL uptake; *P2ry6* receptor gene knockout has been reported to inhibit atherosclerotic lesion progression (58). Similarly, sustained activation of the Gsα subunit has been shown to upregulate CD36 and SR-A expression via the cAMP/CREB pathway, thereby enhancing lipid uptake and foam cell formation (59). In contrast, NFATc3 has been proposed to exert a protective effect by transcriptionally upregulating miR-204, which may suppress SR-A and CD36 expression and limit macrophage foaming (60). Furthermore, Siglec-E has been reported to inhibit ox-LDL uptake by interacting with CD36 and suppressing downstream VAV signaling (61). Notably, these inflammatory receptors may also influence cholesterol efflux pathways. Studies have suggested that blocking Toll-like receptor signaling can restore ABCA1, ABCG1, and LXRα/PPARγ expression by inhibiting NF-κB and MAPK (p38, c-Jun N-terminal kinase) activity, thereby reestablishing the cholesterol efflux program in macrophages (62) (Fig. 4).

In addition to transcriptional regulation, posttranslational ubiquitination of transporters and receptors represents an additional layer of control over the balance between lipid uptake and efflux. In this context, Scavenger receptor class B type 1 (SR-B1) should be viewed as a context-dependent regulator of macrophage cholesterol homeostasis rather than solely as an uptake-related receptor. Previous studies have shown that SR-B1 contributes to macrophage sterol efflux, particularly in human macrophages, where it facilitates cholesterol efflux to HDL (63). Consistent with this, functional neutralization of SR-B1 or the presence of loss-of-function SCARB1 variants has been associated with reduced cholesterol efflux capacity (64). However, recent evidence suggests that, in specific pathological microenvironments, SR-B1 may also participate in lipid uptake. Studies have proposed that the E3 ubiquitin ligase RNF128 stabilizes SR-B1 through Lys63-linked ubiquitination, thereby preventing its lysosomal degradation; sustained retention of SR-B1 at the PM may subsequently promote ox-LDL uptake and accelerate foam cell formation (65). Together, these findings suggest that ubiquitination-dependent regulation of receptor stability may reshape the functional balance of SR-B1, allowing the local plaque microenvironment to disturb macrophage lipid homeostasis through multiple layers of protein turnover.

### ER stress and activation of the NLRP3 inflammasome

ER stress is increasingly recognized as an important link between macrophage lipid overload and lipotoxic inflammatory signaling. Persistent ER stress may impair cholesterol efflux partly by suppressing LXRα-dependent transcription and reducing the expression of ATP-binding cassette transporters ABCA1 and ABCG1. Concurrently, it may activate stress-related signaling pathways such as c-Jun N-terminal kinase, thereby promoting pro-inflammatory macrophage polarization and accelerating foam cell progression (6, 66). In microenvironments exposed to ox-LDL and hyperglycemia, ER stress and oxidative stress can reinforce each other, which may further compromise ABCA1-dependent cholesterol homeostasis and aggravate intracellular lipid droplet accumulation (24, 66).

Recent studies suggest that when cholesterol efflux is insufficient, excess PM free cholesterol may be redistributed to the ER through non-vesicular transfer mechanisms at membrane contact sites, potentially contributing to ER cholesterol overload and facilitating NLRP3 inflammasome assembly and Caspase-1 activation (67). This process may involve Aster-family proteins, although the precise molecular details remain under investigation.

This mechanism has been supported by both genetic mouse models and human observational evidence. Studies have confirmed that myeloid-specific double knockout of Abca1 and Abcg1 activates the NLRP3 inflammasome and promotes neutrophil extracellular trap formation, thereby accelerating the progression of necrotic cores within atherosclerotic plaques (19). This systemic immune mechanism, initially identified in mice, has been corroborated by human data: patients with Tangier disease, caused by loss-of-function mutations in ABCA1, have been reported to exhibit elevated plasma levels of IL-1β and IL-18 (19). These findings suggest that the failure of the macrophage cholesterol efflux program may contribute not only to lipid accumulation but also to the amplification of innate immune inflammation.

### Autophagy-lysosome dysfunction

The autophagy-lysosome axis is an important endogenous degradation pathway through which macrophages clear intracellular lipid loads and maintain lipid homeostasis. Emerging evidence suggests that lipophagy participates in the lysosomal degradation of lipid droplets and is closely associated with cholesterol efflux and lipid homeostasis (68). By promoting the delivery of lipid droplets to lysosomes for hydrolysis, lipophagy may facilitate the generation of free cholesterol and its subsequent efflux; this process is functionally coupled with ABCA1/ABCG1-mediated cholesterol efflux and may attenuate lipotoxicity-associated inflammatory responses. As AS progresses, persistent lipotoxic and oxidative stress can impair autophagy-lysosome function, leading to disrupted autophagic flux and reduced lipid clearance capacity (68).

The pathological basis of this autophagy-lysosomal dysfunction can be summarized as impaired autophagosome-lysosome fusion or maturation, reduced lysosomal acidification, and decreased lysosomal hydrolytic capacity, ultimately leading to incomplete degradation of intracellular lipid substrates. When the load of oxidized lipids or oxysterols exceeds the cellular processing capacity, lysosomal membrane damage and dysfunction may occur, accompanied by decreased activity of acid hydrolases, thereby further impairing lipid degradation capacity (40, 68). Impaired lysosomal function can further hinder autophagosome maturation and fusion with lysosomes, resulting in the accumulation of autophagy-related vesicles and the formation of autophagic flux blockade (68, 69). In addition to lysosomal damage, abnormalities in autophagy initiation may also contribute. Studies have suggested that downregulation of the Beclin1/Class III PI3K axis and aberrant activation of PI3K/Akt/mTOR signaling may together suppress autophagy initiation and exacerbate lipid metabolic disturbances (69, 70). These signaling abnormalities may be accompanied by impaired lipid droplet hydrolysis and reduced ABCA1/ABCG1 expression, thereby compromising cholesterol efflux capacity and promoting intracellular lipid accumulation and foam cell formation.

This stress-induced impairment of lipophagy has been validated in in vitro foam cell models and is associated with macrophage lipid accumulation, foam cell formation, and enhanced inflammatory responses in atherosclerotic plaques (40). Therefore, maintaining the integrity of the autophagy-lysosome system and restoring the functional coupling between lipophagy and cholesterol efflux may represent an important strategy for intervening in lipid metabolic imbalance in AS.

### Pathological remodeling of RCT by systemic inflammation

In addition to local inflammatory activity within atherosclerotic plaques, systemic inflammatory stress may also modulate the efficiency of RCT by altering the composition and functional properties of circulating lipoproteins. Experimental studies have indicated that intense stressors, such as surgical stress, can induce inflammatory remodeling of circulating HDL particles, characterized by increased enrichment of acute-phase reactants such as SAA1/2 and a concomitant reduction in ApoA-I abundance [56]. Such compositional changes may impair HDL function and thereby reduce its capacity to support macrophage cholesterol efflux, ultimately compromising RCT efficiency (10). Moreover, available evidence suggests that different foam cell subtypes may respond differently to inflammatory stimuli: surgical stress appears to exert a more pronounced inhibitory effect on RCT in macrophage-derived foam cells than in VSMC-derived foam cells, implying distinct susceptibility to systemic inflammatory insults across foam cell populations (10).

Inflammatory remodeling of circulating HDL may also weaken its protective functions during the plasma transport phase of RCT, including its cholesterol acceptor capacity and anti-inflammatory activity. Through these changes, systemic inflammation may reduce the efficiency of cholesterol removal from plaque foam cells and thereby contribute to impaired lipid clearance from atherosclerotic lesions. Collectively, these findings support the view that systemic inflammation can promote pathological remodeling of the RCT pathway and may thereby facilitate atherosclerotic progression under stress conditions.

## Neurofoam cells and lipid metabolic disorders in SCI

### Molecular mechanisms of myelin fragment overload and foam cell formation

Following SCI, the rate of myelin fragment production during acute demyelination often exceeds the processing capacity of the endogenous phagocytic system, leading to the accumulation of cholesterol-rich myelin debris in the injury microenvironment (71). As a metabolic burden, the persistent accumulation of myelin debris is considered a key factor that induces the transformation of macrophages/microglia into a foam cell phenotype, thereby sustaining chronic inflammation and inhibiting axonal regeneration (5, 72). Macrophage uptake of myelin fragments is not a simple clearance process, but rather a metabolically programmed event finely regulated by multiple scavenger receptors; its downstream metabolic fate largely determines whether the post-SCI immune microenvironment shifts toward repair or exacerbation of injury.

Based on receptor-mediated clearance efficiency and metabolic preferences, relevant receptor pathways can be broadly categorized into distinct metabolic axes. The phagocytic pathway, represented by MSR1/SR-A1, may exhibit a decoupling of uptake and metabolic capacity following SCI: although MSR1 can efficiently mediate myelin endocytosis, cholesterol may accumulate abnormally within the cell due to limitations in the compensatory capacity of intracellular cholesterol transporters (such as ABCA1/ABCG1). This metabolic stasis may further activate NF-κB-mediated pro-inflammatory signaling and promote M1-like polarization, thereby inducing the release of neurotoxic mediators (73). In contrast, receptor pathways such as TREM2, Mertk, and Axl are more involved in the maintenance of lipid homeostasis. TREM2 activation may not only accelerate clearance kinetics by enhancing phagocytic efficiency but also promote remyelination by reshaping cholesterol metabolic flux (74, 75, 76). Furthermore, CD36 may exhibit context-dependent bidirectional metabolic regulation in this pathological setting, potentially contributing to feedback regulation of lipid load through activation of the PPAR/LXR axis (77).

In addition to direct receptor-mediated mechanisms, the overall kinetics of myelin clearance are also influenced by the regulation of phagocytic thresholds. The SIRPα/CD47 signaling pathway, acting as a negative phagocytic signal (“don’t eat me” signal), may serve as a regulatory checkpoint that limits clearance rates following SCI (78). Experimental evidence suggests that dynamically downregulating the phagocytosis threshold by blocking such immune checkpoints may accelerate the clearance rate of debris and improve the post-injury repair microenvironment (78, 79). Meanwhile, intervention in the clearance process via the PPARγ-Mertk axis by drugs such as pentoxifylline further suggests that myelin clearance is a dynamic process regulated by multiple signaling pathways (80).

In summary, myelin clearance following SCI cannot be simply dichotomized as “harmful” or “beneficial”; its core pathological feature lies in the dynamic balance between uptake rates and the cell’s capacity for lipid metabolism. When the rate of myelin uptake mediated by the aforementioned scavenger receptor axis exceeds the threshold of the cell’s active efflux and hydrolytic metabolism, macrophages may enter a state of lipid overload and gradually transform into foam cells that drive secondary damage in SCI. This metabolic imbalance is not only a direct consequence of myelin accumulation but may also constitute a pathological basis for the aforementioned dysfunction in autophagy and cholesterol efflux.

### Macropinocytosis as a pathological mechanism underlying dysregulated lipid uptake

In addition to receptor-mediated uptake pathways, macropinocytosis represents an important receptor-independent mechanism that contributes to dysregulated intracellular lipid acquisition in the lipid-rich microenvironment after SCI. As a high-capacity, actin-dependent endocytic process, macropinocytosis can expand the amount and diversity of extracellular material internalized by macrophages beyond the limits imposed by receptor abundance and ligand specificity (7). This feature may allow macrophages to acquire lipids beyond their metabolic handling capacity under pathological stress conditions.

This high-capacity uptake pattern may have distinct pathological consequences during SCI-related foam cell formation. Because macropinocytosis involves bulk uptake of extracellular fluid through membrane ruffling and vesicle formation, macrophages may internalize myelin debris and other non-myelin lipid-containing material distributed within the injury zone (5, 7). Experimental evidence indicates that blocking receptor-mediated endocytosis does not fully abrogate foam cell formation, supporting the contribution of receptor-independent uptake pathways, including macropinocytosis, to the total lipid burden after SCI (7). Sustained uptake of heterogeneous lipids can rapidly overwhelm macrophage lipid-processing capacity (81).

Excess neutral lipids and free cholesterol internalized through macropinocytosis may exert pro-inflammatory effects during intracellular processing. Recent SCI evidence shows that macropinocytosis-associated lipid overload is accompanied by intracellular cholesterol crystal formation (7). These cholesterol crystals may contribute to the persistence of the foamy phenotype and can act as danger-associated signals that promote ROS generation and NLRP3 inflammasome activation, thereby enhancing downstream pro-inflammatory cytokine production (7). Overall, under pathological SCI conditions, macropinocytosis may amplify lipid overload in concert with receptor-mediated pathways and contribute to macrophage dysfunction and the acquisition of a neurotoxic phenotype (72).

### Compensatory and decompensatory states of the endogenous lipid clearance network

In response to lipid stress following SCI, microglia and macrophages may initiate a lipid clearance program regulated by nuclear receptors such as LXR, PPAR, and RXR (72, 82); however, this network may become functionally decompensated under sustained pathological stimulation (53). In the CNS under physiological conditions, APOE and ABCA1/ABCG1 constitute a key clearance network that maintains the balance of local lipid dynamics (13, 15, 17). Bioinformatics and transcriptomic evidence indicates that APOE is a hub gene associated with transcriptional programs linked to lipid transport, autophagy, and inflammatory responses in macrophages and microglia after SCI (17). Under the lipid-laden conditions induced by traumatic injury, the reactive upregulation of endogenous APOE may represent a compensatory response that helps limit excessive inflammation (17).

However, the local increase in ROS following SCI may disrupt this clearance network (51). Persistent pathological stimulation may contribute to the degradation of ABCA1 on the surface of macrophages through the ubiquitin-proteasome system or lysosomal degradation pathways, thereby impairing cholesterol and phospholipid efflux to extracellular acceptors such as ApoA-I or HDL particles (46, 53). The impairment of cholesterol efflux may be accompanied by reduced efficiency of the lipophagy-lysosomal hydrolysis pathway, which mediates the turnover of intracellular lipid droplets, thereby further limiting intracellular lipid turnover (40). This disruption of autophagic flux may lead to the accumulation of neutral lipids as lipid droplets or lipid degradation intermediates, potentially contributing to a pro-inflammatory cycle characterized by impaired efflux, progressive lipid accumulation, and oxidative stress (68, 70). Although endogenous miR-223 or RXRα may participate in the transcriptional regulation of ABCA1 to maintain sterol turnover, this protective mechanism may be blocked by subsequent inflammatory signaling pathways, potentially contributing to long-term foam cell retention (72, 82).

### Multidimensional glial cell interactions: a topological regulatory network for local lipid homeostasis

Lipid metabolic imbalances in SCI or CNS demyelinating lesions are not driven by a single cell lineage alone, but are governed by a local lipid homeostasis regulatory network constructed through the interwoven interactions of diverse glial cell lineages (83). In a chronic cerebral ischemia white matter injury model, reactive astrocytes can release the chemokine CXCL5, which acts on local microglia via CXCR2, inhibiting their phagocytosis and processing of myelin debris, thereby aggravating white matter injury and impairing myelin clearance (84). On the other hand, the local lipid buffering capacity in the injury microenvironment is regulated by heterogeneous paracrine interactions among different glial cell lineages. A 2026 Nature study showed that reactive astrocytes in specific white matter injury states can secrete the matricellular protein CCN1, which modulates microglial lipid metabolism through an SDC4-dependent mechanism. Activation of the CCN1–SDC4 signaling axis can remodel microglial lipid metabolism, enhancing lipid droplet formation and lipid buffering capacity while reducing cholesterol efflux, thereby promoting efficient clearance and metabolic turnover of phagocytosed myelin debris. This process may help establish a protective cellular state at the injury site that limits the spread of lipotoxicity and supports white matter repair (83, 85). Furthermore, the lysosomal processing, cholesterol esterification, and lipid efflux of myelin fragments by microglia may, through metabolic coupling, support the local lipid environment required for oligodendrocyte differentiation and remyelination; however, whether this process directly drives the synthesis of lipid precursors in astrocytes or oligodendrocytes requires further investigation (85).

In summary, the pathological evolution of foam cells in the lesion microenvironment and the decompensation of lipid turnover are governed by intercellular crosstalk among astrocytes, resident microglia, and peripherally infiltrating macrophages across multiple dimensions, including myelin processing, lipid droplet degradation, and repair signaling. Therefore, to elucidate the mechanisms underlying lipid metabolism disorders in SCI, it is essential to transcend the limitations of research focused on a single phagocytic lineage and instead place these processes within the context of this dynamic glial-immune topological network for a systems biology analysis.

In summary, while macrophages in AS and microglia in SCI share overlapping cascades of lipid overload, they exhibit distinct organ-specific variations in their microenvironmental triggers, nonspecific phagocytic pathways, and glial-immune topological networks. A multidimensional and comprehensive comparison of the molecular networks governing foam cell formation between these two diseases is systematically summarized in Table 1.

**Table 1:** Multidimensional comparison of cellular and molecular networks governing foam cell formation in atherosclerosis and spinal cord injury

Dimension	Atherosclerosis (AS)	Spinal Cord Injury (SCI)	Common Mechanism
Main lipid source	ox-LDL and modified lipoproteins	Myelin debris, membrane lipids, and injured tissue lipids	Excess lipid input
Main phagocytic cells	Macrophages and VSMC-derived foam cells	Macrophages and microglia	Increased lipid load after phagocytosis
Uptake pathways	CD36, SR-A, SR-B1, P2Y6R, Gs alpha, NFATc3, RNF128, etc.	CD36, Axl, Mertk, TREM2, CR3, SIRPα/CD47, MSR1, and macropinocytosis	Enhanced or imbalanced uptake
Efflux pathways	ABCA1, ABCG1, apoA-I, and LXR	ABCA1, ABCG1, APOE, RXRα, PPARγ, and miR-223	Insufficient cholesterol efflux
Intracellular impairment	ER stress, impaired autophagy, ABCA1 degradation, and lipid droplet accumulation	ER stress, autophagy/lysosomal impairment, PLIN2, UBE3A, and lipid droplet accumulation	Failed lipid processing
Inflammatory consequences	NLRP3, NETosis, and plaque instability	NLRP3, IL-1β, TNF-α, neuronal apoptosis, and impaired white matter repair	Sustained inflammation
Therapeutic directions	LXR/RXR agonists, ABCA1 stabilizers, anti-inflammation, and enhanced RCT	Enhanced myelin clearance, ABCA1/APOE/RXR axis, and pro-repair immune reprogramming	Cross-disease metabolic-inflammatory intervention

## Therapeutic strategies targeting the remodeling of macrophage lipid metabolism

### Mechanism-driven pharmacological interventions and natural multitarget compounds

By targeting different pathological disruptions in the macrophage lipid turnover pathway, various natural phytochemicals and compound mixtures have demonstrated potential due to their multi-target synergistic effects. Restoring upstream lipophagy is considered a prerequisite for resolving downstream efflux blockages (Fig. 5, Layer 1). For example, the antioxidant Mito-Tempol can restore autophagy flux by scavenging ROS in the microenvironment, thereby reversing lipid accumulation and promoting ABCA1/ABCG1-mediated cholesterol efflux (68) (Fig. 5, Layer 1). At the level of traditional Chinese medicine formulations and individual compounds, certain traditional Chinese medicine preparations have been reported to reduce lipid deposition in macrophages by enhancing autophagy (70). Mangiferin combined with an LXR agonist (T0901317) has been shown to achieve synergy between autophagy induction and cholesterol efflux via the AMPK signaling pathway (86) (Fig. 5, Layer 1 and 3). Once lipids enter the processing phase, maintaining the stability of efflux channels becomes a key determinant of turnover efficiency (Fig. 5, Layer 2). Given that efflux channels are susceptible to ubiquitination or lysosomal degradation under pathological conditions, current intervention strategies have shifted from a focus on “transcriptional upregulation” to “inhibiting target protein degradation and prolonging effective half-life.” In this regard, piperine can block the degradation of ABCA1, potentially through inhibition of calpain-mediated proteolysis (47) (Fig. 5, Layer 2). Apolipoprotein A-I binding protein can inhibit foam cell formation by stabilizing the ABCA1 protein complex (49). Furthermore, the dietary component falcarindiol has been reported to possess dual functions: activating ABCA1 gene transcription and maintaining ABCA1 protein stability (48).

**Fig. 5:**
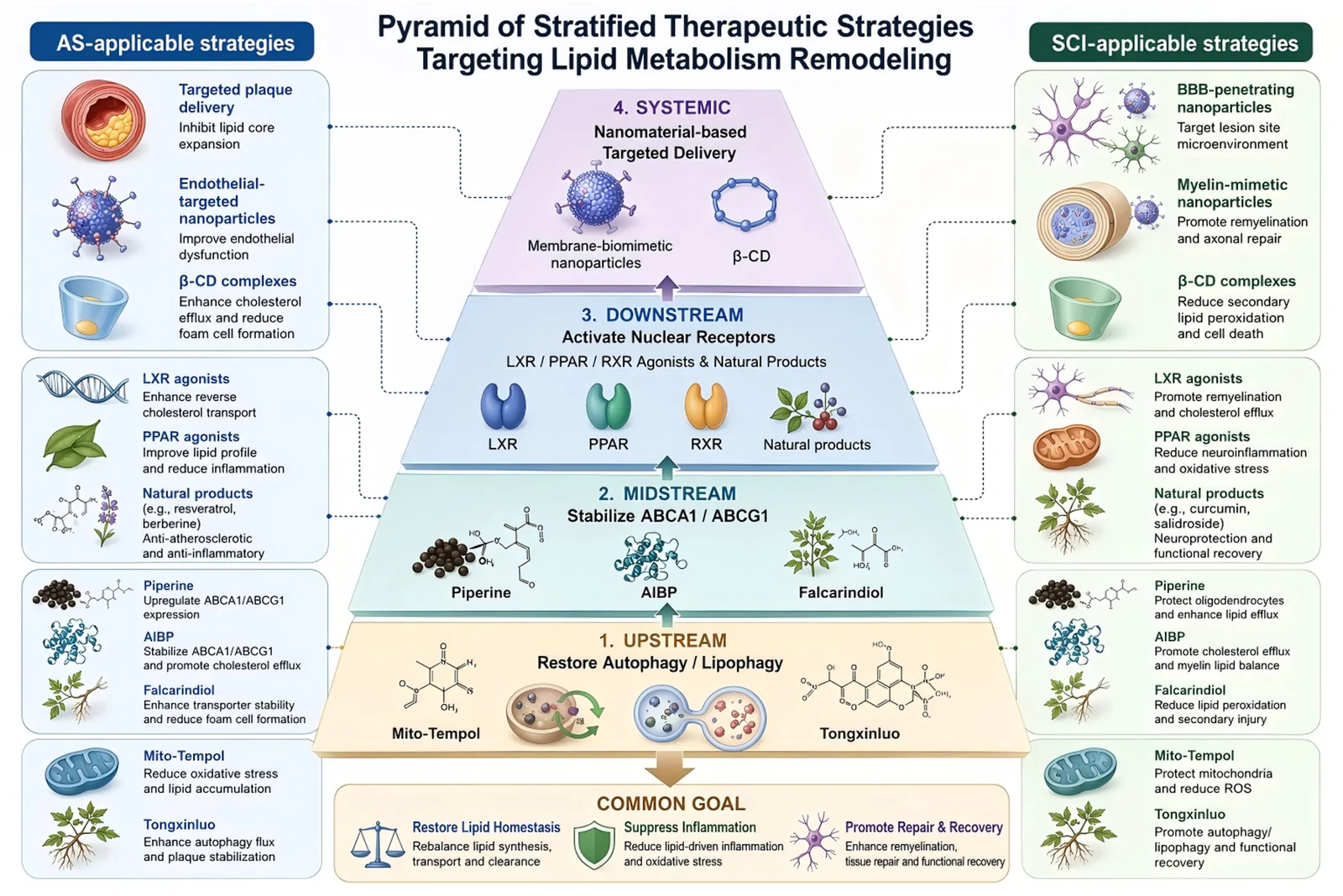
Stratified therapeutic strategies targeting macrophage lipid metabolism remodeling. Pyramid diagram summarizing four layers of intervention strategies. Bottom layer (upstream): restoration of autophagy and lipophagy using agents such as Mito-Tempol and Tongxinluo. Second layer (midstream): stabilization of ABCA1/ABCG1 efflux channels using piperine, AIBP, and falcarindiol. Third layer (downstream): activation of nuclear receptor transcriptional programs (LXR/PPAR/RXR) using synthetic agonists and natural products. Top layer (systemic): nanomaterial-based targeted delivery using membrane-biomimetic nanoparticles and β-cyclodextrin (β-CD). Left side: strategies applicable to atherosclerosis (AS). Right side: strategies applicable to spinal cord injury (SCI). Bottom: common therapeutic goal—restore lipid homeostasis, suppress chronic inflammation, and promote tissue repair. ABC, ATP-binding cassette.

At the broader level of transcriptional regulatory networks, targeted activation of the LXR/PPAR/RXR nuclear receptor axis is a classic pathway for driving RCT(Fig. 5, Layer 3). Perilla extract has been shown to upregulate ABCA1/ABCG1 expression via the PPARγ/LXRα axis, thereby enhancing lipid efflux (31, 32, 87). Notably, such multi-target natural products may achieve bidirectional regulation of lipid and immune pathways. For example, mulberry extract has been reported to upregulate ABCA1/ABCG1 and may inhibit p38 MAPK-NLRP3-mediated inflammatory responses (34) (Fig. 5, Layer 3). Furthermore, the efficacy demonstrated by quercetin in improving metabolic syndrome models suggests that the dynamic “cholesterol efflux capacity” of macrophages may hold greater clinical value as a functional endpoint than static HDL-C concentration (88, 89). In combination with emerging epigenetic and metabolic regulatory approaches—such as targeted activation of the SIRT1 pathway using honeysuckle polyphenols (90), or the use of engineered vectors to deliver anti-miR-33a-5p to relieve the transcriptional repression of ABCA1 (91)-these mechanism-driven intervention networks collectively provide a pharmacological foundation for reshaping localized lipid homeostasis in both central and peripheral systems(Fig. 5, Layer 3).

### Biomimetic nanodelivery systems and spatial targeting in the microenvironment

Although systemic activators of lipid metabolism can promote cholesterol efflux and RCT, their clinical translation is often constrained by off-target effects and organ-specific toxicity, including potential hepatic lipid perturbation. In this context, biomimetic nanotechnology is shifting lipid-metabolism intervention from broad systemic exposure toward lesion-oriented delivery and spatially restricted action (Fig. 5, Layer 4). By exploiting the molecular resemblance between foam cells and the cellular components present in atherosclerotic plaques or injured spinal cord tissue, membrane-camouflaged nanoplatforms can enhance lesion accumulation through homotypic or inflammation-guided targeting. For example, platelet-membrane-fused exosome-like nanovesicles can precisely homing to lipid plaque lesions, targeting the upregulation of ABCA1/ABCG1 expression (92) (Fig. 5, Layer 4); nanoparticles mimicking the macrophage membrane can inhibit lesion expansion by synergistically enhancing efferocytosis and cholesterol efflux (93) (Fig. 5, Layer 4); and a MnO_2_ nanosystem functionalized with macrophage membranes has achieved a triple ecological remodeling by clearing ROS in the microenvironment, alleviating local hypoxia, and promoting lipid turnover (94) (Fig. 5, Layer 4). Collectively, these studies support the concept that biomimetic membranes can improve local delivery specificity while coupling anti-inflammatory effects with lipid turnover.

β-Cyclodextrin, owing to its cholesterol-complexing and extraction properties, has also been explored as a local lipid-remodeling component in nanoplatform design. When incorporated into lipid-based delivery systems, β-Cyclodextrin may enhance the mobilization and solubilization of local cholesterol, but its therapeutic contribution, dose window, and safety profile require further validation in disease-specific models (95) (Fig. 5, Layer 4). For SCI, biomimetic nanoplatforms may offer a strategy to cross or partially traverse the blood–spinal cord barrier and concurrently address myelin debris accumulation, inflammasome activation, and dysregulated lipid handling in the lesion core (Fig. 5, Layer 4). However, the therapeutic window, white-matter selectivity, pharmacokinetics, biodistribution, and long-term metabolic toxicity of such systems in the CNS remain to be systematically evaluated.

### Cutting-edge evidence and theoretical closure for cross-disease translation

Recent translational evidence has strengthened the mechanistic rationale for the proposed “peripheral-to-central lipid-handling convergence” between AS and SCI. In a 2026 study, acute postoperative inflammatory stress was sufficient to rapidly remodel the HDL proteome, reduce cholesterol efflux capacity, and impair RCT in *Apoe*−/− mice, with parallel evidence of reduced efflux capacity in postoperative human plasma (10) (Fig. 5, Layer 3). The impairment was more pronounced in macrophage RCT than in VSMC RCT, indicating that cholesterol handling is highly dynamic and cell-type sensitive to microenvironmental stress. These findings reinforce the view that lipid efflux and RCT are not static housekeeping functions but stress-responsive processes that can be rapidly disrupted by inflammatory cues.

In SCI repair, emerging evidence suggests that effective recovery depends not only on increasing phagocytic uptake, but more critically on improving the intracellular processing, lysosomal maturation, and detoxification of internalized myelin debris (96) (Fig. 5, Layer 1). A recent study identified the FGF4/FGFR1–PI3K/AKT–Clec10a axis as a regulator of macrophage-mediated myelin debris clearance: FGF4 enhanced phagosome maturation, restored endolysosomal degradative capacity, reduced lysosomal membrane permeabilization, attenuated NLRP3 inflammasome activation, and promoted a reparative macrophage phenotype (72, 81) (Fig. 5, Layer 1). The multidimensional convergence of the previous evidence collectively establishes the core argument of this paper: local lipid buffering and turnover capacity are the most critical molecular determinants governing the transition of the traumatic microenvironment from a pro-inflammatory toxic phenotype to a tissue-repair-promoting phenotype.

## Conclusions

In summary, irreversible decompensation of macrophage lipid homeostasis is not an isolated disease phenotype, but rather a common pathophysiological basis underlying both AS and SCI; the core feature of both is persistent pathological lipid overload that ultimately exceeds the compensatory limits of intracellular endogenous processing and efflux. This pathological homology—spanning substrate overload (ox-LDL or myelin debris), cholesterol crystallization, and sustained NLRP3 inflammasome activation—provides profound mechanistic feasibility for translating strategies for reshaping RCT in the vascular field to the repair of neural trauma. Although systemic activation of lipid efflux (e.g., via conventional LXR agonists) has long been limited by off-target toxicities such as hepatic steatosis, precise intervention strategies—including cell-specific nanotargeted delivery, highly selective receptor modulation, and posttranscriptional stabilization of ABCA1/ABCG1—hold promise for thoroughly restructuring local lipid homeostasis while circumventing the risks of systemic lipogenesis. In the future, research on cross-organ inflammatory diseases urgently requires a paradigm shift from “single-pathway intervention” to “remodeling of the microenvironmental ecosystem”; Leveraging the deep integration of single-cell and spatial multiomics (transcriptomics and lipidomics) technologies to comprehensively analyze the lipid metabolic heterogeneity and spatiotemporal trajectories of phagocyte foam formation within lesions will provide the theoretical foundation for establishing a precision medicine intervention framework covering the entire “uptake-processing-efflux” chain. Understanding and reconstructing this impaired lipid metabolic foundation will undoubtedly become a universal new engine for translational medicine in the future, breaking through the barriers of chronic inflammation and driving endogenous tissue regeneration.

## Conflict of interest

The authors declare that they have no conflicts of interest with the contents of this article.
